# Turkey from a Medical Point of View

**Published:** 1861-05

**Authors:** W. Goodell

**Affiliations:** Member of the Imperial Society of Medicine at Constantinople


ART. III.—*Turkey from a Medical point of View.* By W. GOODELL, M.D., member of the Imperial Society of Medicine at Constantinople.

ALTHOUGH the justly distinguished names of Rhazes, Avicenna, and Lockman are familiar as household words to the Mohammedan, yet the Turks, who cheerfully adopted the religion as well as the literature of the Saracens, when the Ottoman Empire absorbed the conquests of the Caliphs, seem in nowise to have encouraged the study of the medical or surgical sciences which flourished so brilliantly among the Arabians in the ninth and tenth centuries. In place of advancing, they have retrograded to the low ebb of the dark ages, and, as in that period, the remedial art is monopolized by the Jewish leech, and by strolling mountebanks and juggling impostors. In the larger sea-ports, where civilization and polished manners are aimed at, there are many very talented physicians, whom political troubles, or a spirit of adventure, have driven to the East; and they deservedly rank high in the esteem of the more enlightened inhabitants. But in the vast majority of cases, either native empirics are employed, or else Greek and Italian apothecaries, who, too ambitious to limit their sphere of usefulness to the mortar and pestle, retail the well-conned prescriptions of the more regular practitioner.

In the palmy days of the Janizaries, military surgery was not a whit better. After a gunshot wound, there was no caviling about the niceties of shock and reaction, but a council of officers determined the necessity of an operation, and an amputation depended upon the decision of the chaplain; consequently, most of the wounded perished; indeed, it is a notorious fact that even after disastrous wars, no mutilated warriors encumbered the finances of the state. At length the Turkish government, becoming keenly alive to the importance of educated attendants on their armies, established in Constantinople, about thirty years ago, a university of medicine, similar to those of Europe. Richly endowed, it not only gives gratuitous instruction to about five hundred youths of all religions and nationalities, but each student, upon his entrance, receives a military rank with corresponding rations; which are increased to those of a higher appointment at the end of the course, when he is bound to serve five years in the army. The course of instruction cannot be the most thorough, especially since the knowledge of anatomy must be derived from the study of manikins and of the inferior animals. The Koran does not sanction human dissections, assigning as a reason that contact with a dead body renders a Mohammedan unclean, and that the soul does not forsake the body immediately after death, but lingers until the moment of interment. Hence, the corpse is hurried to the grave as rapidly as the bearers can go, and post-mortem investigations are popularly invested with all the horrors of human vivisections. Recently, the authorities have connived at the yearly dissection of two or three negro galley-slaves; but we have met graduates of ten years standing who had never examined the interior of the human frame, and whose topographical ignorance of anatomy would insure a flogging to the pupils of one of our public schools. The occupants of the various chairs of this institution, with some honorable exceptions, are ubiquitous Greeks and Armenians of no mark, whom successful intrigue or fortunate ties of consanguinity have placed in these lucrative positions. The dean of the faculty rejoices in the soubriquet of head physician, a hereditary title which does not necessitate any knowledge of medicine; the present incumbent not knowing the difference between the pons Varolii and the pons asinorum.

During the Crimean war, a royal *firman*, or letters-patent, granted a charter to the Imperial Society of Medicine, at Constantinople, and endowed it with a yearly sum of two thousand dollars. The resident members, numbering about one hundred and twenty, are elected by ballot, upon the presentation of a thesis, which must meet the unanimous approval of a committee appointed for that purpose. This society deserves the highest encomium, and, although its meetings are held weekly, the attendance is better than that of any similar institution of this city. Its library and pathological museum are rapidly increasing; it supports a reading-room which is open every day to members, and publishes a very excellent monthly gazette, the editors of which are elected every six months. The debates, held usually in the French language, are of a most instructive and interesting character, inasmuch as disputants of different nationalities, and consequently disciples of every known European school, alternately defend and challenge the doctrines of rival teachers.

Although the marked improvement in the education of the upper classes of Turks impels them to seek the advice of educated physicians, yet, whenever the disease is one of doubt, or unusually protracted, there is an instinctive tendency to call in the impudent quack, who deals in nostrums or incantations, according to the religious or secular character he prefers to assume. Even the Sultan, who may be considered as a fair type of the liberal Mohammedan, is not free from this foible, and rarely employs the services of his seven Christian court-physicians, although each is obliged in turn to spend twenty-four hours of every week within the walls of the palace. Among the score of empirics of every sex and sect, who hang about the precincts of the seraglio, there are two or three who rejoice in an ephemeral pre-eminence, which they retain so long as their remedies meet with success. These may more properly be called the medical attendants of the Sultan.

At one time, Abdul-Mejid conceived a great attachment to an Austrian physician, whom he consulted on all occasions, but whom he was ultimately compelled to dismiss into honorable exile with the rank of ambassador to a foreign court. For the jealous fears of pashas and chief dignitaries could brook no favorite, who might prove a foreign spy, or successfully expose their intrigues to the royal ear. Shortly after this occurrence, the Sultan, while conning over the Koran, casually discovered a defect in the vision of one eye. Courage is not a characteristic of the commander of the faithful, and in great dismay he sent for one of these empirics, who, either not being familiar with optics, or more probably wishing to make capital, gravely shook his head and recommended certain collyria and the application of leeches. This prognosis was quite sufficient to upset the royal patient; he faithfully commenced the treatment, but experiencing no relief, in great fright telegraphed to Turin for the banished physician.

The latter hastened to Constantinople in a steamboat expressly chartered for the emergency, and, detecting at a glance the nature of the affection, satisfactorily proved to his master that one of his eyes had become more presbyoptic than the other. The treatment consisted simply in the substitution of a more powerful lens, while a splendid city residence was the reward of the fortunate oculist.

Upon another occasion, the aristocratic nerves of his majesty were cruelly unstrung by the vulgar twinges of a toothache; which, imitating the example of many a rebellious pasha, traitorously entrenched itself within a molar, and refused to capitulate to the innumerable ptisans, fomentations, nostrums, spells and incantations of the whole empirical and non-empirical faculty. Spurning the tears of the Sultanas, and the entreaties of all the favorite *Odaliques* of the *harem*, the royal sufferer sleeplessly paced his ancestral halls for several days and nights, before he could screw up his courage to the pulling point. But, to the dismay of the chamberlain, in all Stambul not a barber or dentist, for love of money or reputation, was found so disloyal as to aspire to the *crown* of his imperial sovereign. American dentists may smile at the pusillanimity of their Oriental brethren, but let them remember that any accident in the extraction of a royal tooth, whether fracture, delay, or any additional pain, might consign the bold operator to the bastinado, or to the tender mercies of the bowstring. At last, an obscure Jew, who had never looked higher than the jaws of his Hebrew customers, was induced to risk his heels and his neck in the dental encounter. Thrice prostrating himself, he entreated the Sultan to show his slave the offending molar. Quick as thought the forceps were applied, and immediately the Jew fell down, with a piercing shriek, at the feet of his master, in a well-assumed fit of epileptic convulsions. Up jumped the Sultan from his throne, forgetting, in his terror, his toothache, his dignity, and the pain of the extraction, and ordered his pages to bring cordials and water for the unfortunate dentist. The wily Jew, perceiving that a hydropathic treatment was imminent, and that this buffoonery had produced the desired effect of distracting the royal attention, now convalesced with great promptness, triumphantly exhibiting the tooth to the astonished monarch and courtiers. It is hardly necessary to add, that not only was the integrity of his gluteal fascia respected, but Israel went forth from the palace, even unto his kindred, with shekels of gold and shekels of silver.

Two years ago, the Sultan promised himself the pleasure of an extensive trip to all the principal sea-ports of his empire, and preparations were accordingly made, on a scale sufficiently vast for a regular campaign. Neptune, however, proved too staunch a republican to respect the royal stomach, and the descendant of the Caliphs ignominiously beat a retreat to the more obsequious terra-firma of his palace, after remaining in the miasmatic City of Salonica just long enough to acquire a very uncourtly ague. The germs of the disease remained in abeyance, until a tremendous cannonading and pyrotechnic display announced his safe arrival at the metropolis, after a week's absence. That same night, at an hour when all good Mussulmans should be dreaming of bright-eyed houris, an Italian court-physician, who happened to be on duty in the palace, was hastily summoned to quiet the gesticulating limbs of the royal traveler. In spite of the plebeian shakings, rivaling those of this unhappy monarch's still more unhappy ancestor, Bajazet, in his iron cage, our worthy confrere entirely mistook the case. To use a mild expression, he addressed his remedies to the phenomena resulting from an over-dose of champagne; an interdicted beverage which backsliding majesty had freely imbibed, in gratitude for his escape from the gastric eccentricities of the Propontis, and for which he evinces the same partiality that characterized his late brother of Prussia. By good luck, the disease proved a tertian; hence, in spite of the formidable attack of the previous night, the following day found the patient so much improved, that he dismissed his medical attendant with compliments and presents. Four and twenty hours had hardly elapsed, before the court was thrown into confusion by the recurrence of the same train of symptoms. Alas! for the credit of the profession by way of querulous reprisal, an old woman, who enjoyed the reputation of curing the Sultan, in his boyhood, of the small-pox, now ministered to his wants. Knowing her liege lord was a king at the trencher, and consequently attributing the derangement to a postprandial fit of indigestion, she prescribed an aperient, and with her own matronly hands, for "nice customs courtesy to kings," decorously introduced a clyster into the royal person.

Praise be to Allah! the sun dawned on the turban of a convalescent monarch. Quackdom was in its glory. In the first blush of gratitude a pension was settled on the enchantress, while she gathered a rich harvest of Cashmere shawls and jewels from the fair inmates of the palace. By the beard of the thrice blessed prophet, peace to his ashes! again the nocturnal visitor uncourteously shook the royal couch. This time Sultanas, eunuchs, chamberlains, and pages hurried to and fro in the greatest confusion and dismay. Messengers were dispatched for the Grand Mufti, with his formidable train of ecclesiastics, while the whole body politic of cabinet ministers hastened to assemble. After the usual number of pipes and cups of coffee, salaams, pious invocations and quotations, with which every Turk prefaces any important business, the balance of this solemn conclave oscillated now to the partisans of some renowned charlatan, anon to the patrons of the more legitimate art, until it happily depended in favor of science. A well-known Greek physician assumed the treatment, who, to make a long story no longer, boldly unmasked the paludal demon and exorcised it with eighteen grains of quinine.

Fabulous were the rewards of this fortunate conjurer. A noble city mansion, a charming suburban retreat, and a lucrative sinecure evinced the regal gratitude; while, according to Oriental etiquette, the pashas and high functionaries measured their rank by a corresponding value in their gifts to the "man whom the king delighted to honor." The most strait-laced philosophy will not keep down a sigh, when the writer of this article recollects that he was not the dispenser of this scruple of the antiperiodic. A plague on history! it makes us poor plodding mortals envious of our Greek brethren, who have ever been lucky dogs, where good fees are concerned. In all ancient lore, who can find that an obolus, or even an Attic drachma was ever slipped into the expectant palm of an Athenian physician? Such mites were piously consecrated to old Charon's toll, or might secure a back seat in the amphitheatre. Æsculapius undoubtedly set up his chariot and six, and was not only transported from his chair on earth, to a more onerous one among the gods, but received the rich fee of Homeric praise—world-renowned hexameters which we would willingly exchange for a good sterling ante-mortem quid pro quo. Practical Podalirius, "offspring of the healing god," father of phlebotomy, breathes his vows and opens a vein at the same time; and, after this successful venesection, pockets a whole province from the king of Caria and the hand of his daughter to boot. Hippocrates was both aureated and laureated by appreciative Athenians; had his likeness done, not in distemper as was most natural, but in virgin gold by his solid patients of Argos, and had the posthumous honor of temples and altars erected to his memory. Six centuries B.C., enviable Mr. Damocedes, surgeon in ordinary to King Polycrates, and consulting physician extraordinary of all classic valetudinarians, staggers home under the weight of two talents of gold, in exchange for the medical talents he had lavished on the tyrant of Samos; while Darius and his queen, Atossa, without being dunned by a collector, lavished on the same lucky gentleman gifts, honors, and captives innumerable. Precisely as many centuries after the Christian era, Chosroes, a famous king of Persia, grateful for the heroic doses of Stephen of Edessa, rewards that rank abolitionist with the manumission of three thousand slaves. Eheu me miserum! Why can we not trace our genealogy perpendicularly from Cecrops, or legitimately adopt the heraldic device of the Argonautic hero?

But to return from a long digression; the empirics form the vast majority of the medical body, each of whom devotes himself to a specialty, which is hereditary in the family; the father initiating his sons in the mysteries of his craft, and transmitting to his heirs all the secrets appertaining to it. Fractures and dislocations, genito-urinary diseases, hernias, jaundice, epilepsy, etc., are the monopolies of as many different families. The Jews and barbers, who bleed, cup, leech, and extract teeth in addition to their tonsorial duties, are the only general practitioners. They dabble with herbs and simples in the incipient stage of every disease, until alarmed friends apply either to the European physician or empirical specialist; although, as a general rule, the former is considered the dernier ressort, and gains the credit either of the death or the cure. The specialists resort to the most ingenious shifts to inspire confidence in their patients and to disguise their remedies. One gray-bearded, green-turbaned sinner rejoices in being the first oculist of Constantinople. Seated in his box of an office, six feet square, in the most crowded portion of the bazaars, and where every passer-by can witness all proceedings, he prefaces each operation with a long prayer, and administers extreme unction to the golden needle with which he depresses the lens. In connection with this subject, let us not forget a nomadic family of veterinary oculists, who have astonished the intelligent European residents by their success in operating on the cataract of the horse. Arming a curved needle with a stout thread, saturated in some secret liquid, and muttering some unintelligible words, they pass it completely through the lens, leaving the ligature to act as a seton for several hours. We have been assured by many gentlemen, who witnessed this operation in their own stables, that the results were most gratifying.

To appreciate the tricks of those jugglers who deal in spells, some knowledge of the composition of Turkish ink will be necessary. The coloring substance is lampblack, which is thickened and rendered adhesive by gum-arabic. The paper employed is thick and enameled; hence, the characters formed by so viscid a fluid do not readily dry, and every Eastern scribe resorts by necessity as well as by taste to highly-colored sands, the most popular of which are brass-filings and fragments of gold-leaf, thickly sprinkled on, and afterward polished with the smooth convexity of the cypræa. Now the toothache is successfully consigned to the skill of Mohammedan priests, who circumscribe the seat of pain by a circle drawn in an ink, saturated most probably with morphine; within this circumference cabalistic characters are traced, which are eventually to be washed off and swallowed. To the great scandal of the profession in Smyrna, all obstinate cases of intermittent fever, which resisted a regular treatment, resorted successfully to the den of an old *dervish*, a sort of Mohammedan monk, who professed to deal in nothing but incantation. Curious to unfold the mystery, a gentleman, assuming a most unmitigated ague, consulted this pious quack, who, after gravely chanting some stanzas from the Koran, wrote down some well-sanded extracts, from the same holy authority, upon separate slips of paper; ordering each to be placed in a vessel of water over night, and the contents to be drank during the day. It is needless to add that, upon chemical analysis, the sand of our medical caligraphist was found to consist of pure arsenic. In like manner fumigations of cinnabar, and of other mercurial preparations, are adroitly administered under the guise of a voluminous prayer or prolonged invocations. This is a valuable hint which we throw out gratuitously for the consideration of our more ethereal brethren. To the Ozena of the spiritualist or to a recalcitrant node, how efficacious might prove a long message from the shade of the departed Galen, or the ghostly communication from some defunct Ricord!

Another class of empirics, ignoring a religious character, sneer at the divine afflatus, and in their prescriptions confine themselves to whatever is studiedly loathsome. In one case of an enormous cancer of the hip, we found our patient swallowing boluses, whose ingredients somewhat plagiarized the witches' broth in Macbeth, and were as follows:—

Powdered human skull;
Powdered dog's heart;
Dried camel's dung;
Powdered cones of the funereal cypress;
Crude antimony;
Urina virginæ. Q. s.

By the way, this last named rare and precious elixir enjoys a high reputation for curing inflamed eyes, and is as intrepidly taken internally for all hepatic complaints as Congress or Seltzer water. But to return to our patient; on alternate days, the palpitating bodies of ground-rats, eviscerated while alive, and frogs reduced to a pulp in a mortar, were faithfully applied as poultices. In a scientific point of view, this case presented an unusual feature of interest, which we have only observed in one other person, who was dying from hepatic and uterine cancer. A few weeks before death, the whole system seemed so impregnated with the malignant virus that there appeared all over the body hundreds of tumors, from the size of a shot to that of a large walnut, resembling schirrus in hardness and rugosity.

Next come a numerous body of vernal quacks, who, indulgent to popular prejudices, order a venesection in the spring of the year, and prescribe purges and decoctions of frogs and snakes, to eliminate all peccant elements, which appear to hibernate like beasts of prey in high latitudes. Upon the approach of Lent, they also play an important part, by granting plenary indulgence in the good things of this life to such natives as are carnally minded, or have a heretic antipathy to a leguminous diet.

Let us now touch upon a subject as delicately as its nature will permit, and yet which presents great interest in a medical point of view. All Oriental nations have long held the unenviable reputation of being tainted by the same gross licentiousness which caused two ancient cities to be engulphed in the Dead Sea. A few years ago, this vice was openly sanctioned, and every high official not only kept a *harem* of dancing boys, but was publicly surrounded by them on festive occasions. In those days, honorable native families and European residents took the same precaution to preserve the virtue of their sons as the chastity of their daughters. Now, although the Turk may still be justly accused of promoting Priapus, from the guardianship of vineyards to the niches of his Lares and Penates, yet civilization has thrown a veil over its publicity, and the surface traveler would infer a far purer state of morals than actually exists. Absurd as it may seem, there is some force in the argument advanced by the older practitioner, who claims, as a sign of Turkish regeneration, that the proportion of posterior caustic applications are diminishing to the more normal anterior. From frequent conversation with the better class of Turks, who invariably reprehended this custom, we can assign three reasons for its prevalence: The results are more economical; the prurient appetite of the voluptuary craves after novelty; lastly, the scarcity of females, arising from the system of polygamy, by which the wealthier classes monopolize the sex. In addition to this abuse, marriage among the poorer natives is attended with difficulties; for, since a Mohammedan can at pleasure divorce his wife, the latter, to check this license receives a dower from her husband, which she retains after the separation, and it is not every poor man who can afford such a preliminary expense.

The diseases arising from this unnatural relation are purely surgical in their character. We have often treated syphilis disreputably situated, rectitis, hemorrhoids, recto-fossal abscesses, fistulæ in ano, laceration of the mucous membrane of the rectum, prolapsus ani, and relaxation of the sphincter muscles, which were promptly referred by the patients themselves to the proper cause. The latter complaint is so common a one that there is hardly a male prostitute who does not experience more or less inconvenience from it. In two grave cases of rape, laceration of the perineum occurred, in a young Irishman, who was compelled to submit to the embraces of twenty Turkish soldiers, and a lad whose chastity was violated by half a dozen shepherds. The effect on the adult is absolutely negative, excepting in extreme cases, when satyriasis sometimes hideously crowns the career of the worn-out debauchee. It is a remarkable fact, and one based upon the results of long observation, that neither onanism nor pederasty, both of which prevail to a frightful extent in the East, emasculate the *morale* of the Oriental as much as they do the European.

In the former, the physician never witnesses the blushing awkwardness, the unmanly timidity, the restless vision, and the inability to look a stranger in the face, so characteristic of this habit in enlightened countries, which leads us to draw one of two inferences—that either the mental imbecility, resulting from self-pollution in moral communities, should be attributed far more to the humiliation of an educated conscience, than to the constant physical drain on the system; or that where society openly upholds a vice, it is not so apt to be intemperately abused, as when local rules of propriety and the militant precepts of religion compel the votary to invest the indulgence with strict privacy. The secret tippler generally degenerates into the drunkard, while the social glass of a liberal society rarely leads to intemperance.

Since the introduction of New England rum, and the more potent liquors of the Baltic, against whose use ingenious commentators can find no interdiction in the Koran, because they are not the juice of the grape, the far-famed race of Turkish opium eaters is fast dying out. The quiet coffee shops where, twenty years ago, they were wont to assemble, and pass whole hours in ecstatic reverie, now rarely echo with the bubbling of their water-pipes. Alas for romance! that æsthetic vice is rapidly yielding to the more seductive allurements of the glass. But should the traveler become familiar with their haunts, he may now and then see some haggard relic of the old school furtively steal into a quiet corner of the *café*, and, after hastily swallowing a bolus, sink back into a state of dreamy unconsciousness of everything save his chibouque. The celebrated hasheesh is well known, but what is sold in the shops under that name consists of a vile compound of morphine and liquorice. We once had occasion to obtain the analysis of a quantity, to account for symptoms so opposed to the effect of the Indian hemp, and which came nigh terminating the career of a rash American physician. All inveterate opium eaters contend that use so blunts the nervous sensitiveness that large doses of this narcotic simply induce sleep, without previously exalting the fancy. To obviate this fatal deficiency, and to economize the drug, the veteran pleasure-seeker mixes corrosive sublimate with his boluses; and the enormous doses of this poison taken with impunity would exceed all belief, were they not well attested by numerous witnesses. The amount of arsenic taken constitutionally by the mountaineers of Tyrol and Styria are mere pleasantries, compared with the fabulous quantities of the bichloride which these Mithridatean stomachs can tolerate.

To illustrate this fact still further, we will give two anecdotes of a *mollah*, or priest, more celebrated for his devotions to the narcotic than for his genuflections toward Mecca. Entering one day the small drug shop of a Jew, he asked for corrosive sublimate, and, upon its being exposed on the counter, took up a handful, as if to examine the quality, and deliberately swallowed what appeared to be a mouthful. To be accessory to the death of a Turk, however accidental, is a terrible crime; hence, out rushed the Jew from his stall with a howl of terror, showing a clean pair of heels, if so bold a metaphor can be applied to a race which does not take kindly to water. The next morning, disguising himself, with fear and trembling he stealthily approached his shop, peeping around the nearest corner and fully expecting to see it in the hands of the police; but perceiving no signs of unusual excitement, took courage and sat down in his accustomed seat. A few days after, the wily Turk, suspecting the cause of the Jew's escapade, and wishing to turn it to account, entered the door and so worked upon his fears, by threats of prosecution for an attempt to poison one of the faithful, that the latter was only too glad to bribe him into silence.

Flushed with so unexpected a share of the hard-earned savings of an obscure apothecary, our worthy priest determined to try his luck with the far more wealthy druggists of Pera. Entering the store of a Greek, with whom we are well acquainted, he went through with the same manœuvres, swallowing, as the apothecary avers to this day, a piece of the bichloride weighing fully one drachm. After performing this feat, the priest hastily left, declaring the drug was adulterated; while our friend, in an agony of terror lest he should be murdered and plundered by a mob of fanatic priests, who would hasten to avenge the death of their brother, as hastily shut up shop, collected his valuables, and with his family fled into an obscure country town, where he passed a week of dreadful uncertainty. Before venturing to return, he had the good sense to seek the advice of a Turk, who presided over the medical jurisprudence of the realm. This functionary, a veteran opium eater himself, merely smiled, and practically proved to the much relieved apothecary how needless had been his anxiety. Of course the *mollah* returned to make capital out of his feat, but was received so unsuspiciously by the druggist, who coolly asked whether he had been able to procure elsewhere a superior article, that, concluding the game was up, he confessed the object of his second visit, and laughed immoderately over the practical joke he had at first unconsciously played upon the Jew.

The exemption of the dogs of Constantinople from hydrophobia has long been a mystery, and, although not strictly true, is sufficiently so to render their habits an interesting object of inquiry. Resembling a cross between the fox and wolf, acknowledging no master, these city-scavengers form themselves into communities of a dozen or more, and, with surprising intelligence, limit their foraging excursions to certain squares and streets, which are considered their beats and are recognized as such by their canine neighbors. Each tribe has its chieftain, or head-dog, who attains this rank, not by hereditary right, but by power of muscle; whose torn ears, fragmentary tail, and numerous scars attest the veteran warrior—hero of a thousand fights. Although contiguous communities are perpetually at variance and annoyingly quarrelsome, yet offensive and defensive alliances are made by some mysterious pantomime; and when an overwhelming force sweeps down on any district, the four-footed inhabitants of adjacent ones, forgetting past animosities, rush to the rescue with a howl of execration. When one family becomes too large for the offal of its district, some obnoxious member is pounced upon and summarily ejected. With downcast tail, he immediately proceeds to the neighboring district, where, lying on his back, he humbly appeals to the sympathy of the chief-dog, who, with great dignity, approaches the prostrate suppliant. Should his report prove favorable, the fugitive is immediately adopted into the new tribe. On the other hand, should the numerical addition be undesirable, the exile must pass through a perfect gantlet of bites, until he finds a less populous community.

Without shelter of any description whatever, the Turkish dog braves the inclemency of winter with impunity, keeping up the circulation of his blood, during cold nights, by perpetual skirmishes and pitched battles. In the Turkish quarters, fountains are numerous and they can always slake their thirst; but in the European portion of the city, the dog is a much abused animal, often half starved, and often without water for days during the excessive heat of summer. Yet instances of rabies are exceedingly rare, usually occurring among pet dogs, not among those at large. The natives recognize the disease in man, and vaunt as its cure a band of music constantly kept playing to keep the patient awake, in the open air, for the forty-eight hours of the thirty-ninth and fortieth day after the accident. During the twenty-one years of our residence in Constantinople, there occurred but three deaths by hydrophobia, all of which strangely happened in the summer of 1859. Of these three cases, two were clearly traced to the playful bites of pet animals, and the third was one of those exceptional cases, which seem to prove that sometimes the bite of a momentarily enraged dog will produce the characteristic disease.

A Bavarian gentleman, who was infatuated with a morbid desire to destroy every species of dog, in the most brutal and wanton manner, in endeavoring to beat a harmless street dog to death, received a bite from the infuriated animal. The dog had offered him no provocation whatever, and he attributed the fatal attack of hydrophobia, which resulted from the bite, as a just punishment for his former cruelties practiced on those animals.

All these victims were Europeans; because Turks rarely domesticate or abuse the dog; while the last solitary instance of a street dog communicating the disease is not a fair example. With regard to the immunity of the Turkish dog from madness, we incline to the belief that it is owing to the perfect freedom they enjoy in gratifying their sexual appetites; a municipal license which renders the streets of an Eastern city absolutely indecorous in the spring of the year. The orgasm of brutes, observing absolute laws of periodicity and dependent upon the return of certain seasons, must be far more violent than the corresponding permanent instinct in man. The libidinous fury of the stallion is proverbial; while the flesh of many animals not only becomes unfit for food, but sometimes highly poisonous during the period of copulation. Is it not, then, reasonable to infer that any restraint upon so powerful a passion might contaminate the whole system and create a new train of depraved action? This opinion may be founded on error, but it at least possesses some show of plausibility, especially when one considers that almost all instances of rabies occur in pets, whose masters, from various motives, rigorously confine them during heat. There is also another circumstance with regard to Turkish dogs, which possibly may operate in their favor,—the race is pure but plebeian, unmixed with aristocratic blood; while dog-fanciers, in order to multiply varieties and improve breeds, cross and recross the various species, until the original characteristics of each are lost, and their habits become unnatural.

The criminal custom of abortion is universal in all Oriental countries. It is countenanced in the palaces of the great, and practiced in the hovel of the peasant. Since the laws which define the primogeniture of the royal succession are vague and capricious, a mistaken state policy permits every Sultan to destroy his blood relations and the offspring of collateral branches of his family. For example: the present monarch has a brother, several sisters, and three daughters, who have contracted alliances with the aristocracy, but who are prohibited from raising any issue, male or female. Abortive remedies are either employed, or the children strangled at birth by a eunuch who waits in the lying-in chamber for that purpose. Among the upper classes, both polygamy and concubinage encourage abortion as a necessary economical measure. The poor, who always imitate the vices of their superiors, assign poverty as an excuse; until this inhuman practice, adopted by high and low, is not only sanctioned as a custom, but considered a domestic institution and a legitimate necessity.

This operation is usually performed by midwives and Jewish charlatans; although there are regular physicians who chime in with the humors of their patients. Various methods are employed; many smear the os uteri with the impure empyreumatic oil of tobacco which accumulates in their long cherry pipe-stems; others insert a tent of soft chestnut wood, which, swelling from absorbed moisture, is withdrawn to give place to another of larger dimensions. Most generally, however, a rude stiletto is used, with such want of anatomical knowledge that it rarely enters the uterine opening, and not unfrequently penetrates the inferior parietes and fundus of the womb. The fatality of this operation is very great; tetanus and metro-peritonitis hurrying the rash victim to her grave in a few days. The uncertainty of internal medicines renders them less popular; but we have seen two cases of fatal poisoning from the violence of the drugs employed.

At one time, the members of the Imperial Society of Medicine devoted all their energies to purifying this Augean stable of domestic crime, and caused the arrest of one illegal practitioner who had fatally used the uterine stiletto. At first the cabinet ministers promised an active co-operation, but, on sober second thought, pondered over the crying evils of their own large families, and the offender soon emerged from the precincts of the Bagnio with an increased reputation and practice. In the discussion which ensued at one of the sittings of this Society, it was argued that, so long as the Sultan sets the example not only of abortion but infanticide, and so long as polygamy and concubinage are sanctioned by religious antecedents, it would be impossible to enact laws against this unnatural crime. One member arose and stated the case of a nobleman who was blessed with forty-five children, and had recently been deprived of all position of emolument and sent into exile for some alleged misdemeanor. The question was asked, how could the embarrassed finances of such a man support any further increase in his family? The use of the stiletto becomes an absolute necessity. Abortion is no bastard growth, but the natural offshoot of Mohammedanism, and must be coeval with the vitality of Islamism. Other barbarous incrustations may be rubbed off by the constant attrition of intercourse with more civilized nations. But since the paradise of the Moslem embodies an attractive concubinage, which must have its prototype on earth, and since the false prophet quotes divine authority for his Ayesha, Fatima, and Miriam, his followers are bound to uphold polygamy with all its attendant evils.

The commerce in eunuchs is another evil growing out of Mohammed's pernicious matrimonial example. Wives are considered in the light of expensive mistresses, whose constancy must ever be regarded with suspicion; hence the jealousy of the husband requires these sable guardians of the *harem.* This cruel operation is performed during the infancy of Nubian negroes, by Coptic priests, who use no instrument but a razor, and arrest any hemorrhage by the application of hot ashes or the actual cautery. There is a mistaken impression among the profession that simply castration is resorted to. But Turkish jealousy, a keen observer of comparative physiology, marks the amorous proclivities of the ox, and requires complete extirpation of the genital organs. Not only are the testes, but even the scrotum and penis, indeed every vestige of the sex, completely swept away by one stroke of the knife. The urethral orifice is left on a level with the pubes; so that every eunuch is obliged to use a short catheter in order to prevent his clothes from being soiled. The capon, ox, and dray-horse become fat and sleek; but nature abhors the unnatural requirements of the Eastern voluptuary, and stamps the eunuch with hideous ugliness. Not a hair grows on his face; the skin of his forehead and cheeks hangs down in loose folds and wrinkles; his voice resembles the harsh notes of an old woman, or breaks into the discordant key of a lad, who is approaching the age of puberty. His temper becomes irascible, and his disposition most cruel. We have never known a eunuch to be celebrated as a singer; hence we infer that the castration, resorted to in Italy to preserve the fine female voices of boys for ecclesiastical music, could only be successful when the vocal talent had become developed. To add insult to injury, every royal eunuch, according to his rank, has one or more wives, of whom he is proverbially jealous. The Kizlar Agha, or chief eunuch of the palace, ranks as the third dignitary of the empire, and maintains a large *harem*, which is kept under the strict surveillance of subordinate eunuchs. It is a perpetual joke of the court-physicians to compare notes with one another about these women; who, when sick, are so sedulously guarded that the pulse can rarely be touched, and the tongue examined only through a slit in an impervious veil. The career of these females is brief. Vicious practices; sedentary habits; a luxurious table and robes of the thinnest gauze infallibly sow the seeds of consumption. We are not prepared to uphold the author of the "Anatomy of Melancholy" in his assertion that certain esculent roots and rhizomatous vegetables are denied entrance into these establishments; but this fact remains unexplained that, in spite of a liberal supply of coal gas, the weekly allowance of stearine candles for the imperial palace amounts to fifteen thousand pounds.

Small-pox inoculation, which was introduced by Lady Wortley Montague from Constantinople into England, in the earlier part of the last century, is still very much in vogue; but in the larger cities vaccination is becoming more fasionable. In spite of the prevalence of these two methods, the great majority of Orientals are unprotected, and the ravages of this dreadful disease are often terrible. It may justly be considered the only prevailing epidemic of the healthy City of Constantinople. In distant towns of the interior, whose means of communication with the capital are difficult, the want of vaccine matter is often felt; for mothers labor under absurd prejudices, and rarely allow the lymph or scab to be taken from the arms of their children. When out of matter, we have known native quacks make use of tartar emetic, which, upon a raw surface, will produced a pustule highly characteristic of the vaccine virus. The truth of Solomon's apothegm, that "there is nothing new under the sun," is well illustrated by the wild nomadic Kurdish tribes of Asia Minor, who have for centuries practiced vaccination on themselves, invariably taking the lymph from the vesicle of the cow.

Many bigoted Mussulmans, however, object to vaccination and inoculation on religious grounds, and prefer to run the fearful risk of a terrible epidemic, than to yield up one iota of their belief in fatalism. Friends commonly exhibit their sympathy for one another in sickness, by sending their own family physician to the invalid. In accordance with this usage, we were once conducted to the palace of a high Turkish functionary, who had two children, the elder of whom lay alarmingly ill with the confluent form of the disease. A false theology rendered the parents in the end childless; for neither would allow us to prescribe for the sick child, nor vaccinate the other, although tenderly attached to both, and tearfully fearful of the result. Their objections were grounded on a belief that small-pox was a judgment from heaven, and to combat the disease or limit its ravages was a blasphemous endeavor to thwart eternal decrees.

This stern fanaticism and inflexible confidence in fatalism, which make the Turk fearless of the plague, and lead him calmly to smoke his chibouque in a powder magazine, elicit our admiration, however much we may dissent from him. Yet it is often puerile and full of absurd inconsistencies; the doctrine of fate degenerating into a pack-horse which is saddled with human eccentricities. The hands of the physician are often tied by the petulant metaphysics of his patients. A predestinated bleeding may not interfere with the Divine decree, while a draught, particularly if offensive to the taste, shall arouse a holy indignation. In therapeutical theology we have found an emetic, assafœtida, and valerian to be highly heterodox. Clysters and gilded pills, on the contrary, are pronounced canonical by the most peremptory fatalist. When laboring under a predestinated illness, a sincere Mussulman is often harassed with doubts as to the consistency of calling in medical advice. The mooted point to determine is, which physician may be the one predestinated to cure him. To soothe the qualms of a troubled conscience, he consults, at different times, as many different physicians, and obtains from each a separate prescription. All these are next submitted to the nearest apothecary, who, either with prophetic sagacity pounces on the most expensive one, or else, with thrifty ingenuousness, confesses his ignorance of vaticination, and overcomes that deficiency by the ingenious mixture of all the prescribed compounds. After such a display of quibbling logic, one is willing to accept even Cowper's libel, who rhymes the puerile ratiocination of the Moslem with happy humor.

The heroic hygienic treatment of Oriental children would frighten Dr. Combe and cisatlantic mammas. A few days after birth, the spine is scarified in its whole extent, as an outlet for the "bad blood." Salt is then rubbed into the wounds of the screeching infant, and when the complexion becomes cyanotic, through excess of screaming, the palm of the midwife sonorously impinges on the youthful buttock, upon the same principle that a dilatory watch is thumped into increased activity. It is now swaddled up; and its arms and feet so tightly bandaged that all motion, save that of the head, is denied. A cradle is finally prepared into which the child is consigned, and where it remains, from one week's end to another, without being disturbed. An ingeniously contrived glass utensil renders all change of linen unnecessary in the maternal opinion. The mother nurses it by presenting the breast over the cradle; and as it is only washed when the mother is able to go to a public bath, which is often three miles distant, there is no earthly necessity for changing the position of the helpless sufferer. Infantile exercise appears to be one of the unpredestinated items of faith; nothing short of a conflagration will induce the parent to bear her child in her arms. Of course the mortality is frightful, almost rivaling the percentage of foundling hospitals. In this connection, let us observe that, when a new-born infant exhibits no signs of life, the native midwives do not cut the cord, but hasten the delivery of the placenta, which is rapidly transferred to a dish of coals. The only objection we can suggest to this practice is the odor; while it certainly possesses the advantage of not interfering with any other known method of resuscitation. On more than one occasion, we have deliberately pursued Marshall Hall's system of infantile gymnastics; the midwife meantime being scientifically engaged in frying the after-birth on an adjacent brazier.

The Turkish bath is certainly a great luxury, fully deserving the highest encomium. A process so familiar needs no description; but a word or two on its medical virtues may be instructive. Invaluable in chronic rheumatism, in obstinate syphilis, and cutaneous diseases, the friction of the goat-hair gloves and the artistic shampooing render it equally efficacious in dyspeptic complaints. In fatigue, from over mental or physical exertion, nothing can be more grateful; while the disheartening quantities of visible, tangible filth, which peals off from the person of the most scrupulously neat European, do violence to every preconceived notion of cleanliness. Like the far-famed elixir of life, the *Hamam* seems to infuse a glow of youth, a thrill of rejuvenescence into the veins of its most aged votary; although to the novice, fibres of asbestos and a salamander-like nature may seem a welcome property in withstanding its scathing vapors.

Every national custom is liable to some abuse, and the bath, piously restricted to the precincts of a mosque, is often inaccessible to Christian communities. We were once called away to a large Armenian town, about eighty miles from the capital, where no mosque and consequently no bath existed; the inhabitants being obliged to journey a distance of three hours to the nearest Mohammedan village, to enjoy the luxury of an annual ablution. On the other hand, many natives consider two baths per annum as the golden mean; and since the same voluminous suit of clothes and linen are worn unchanged during the interval, the exhalations arising from the Eastern person, however disguised by musk, sadly dis-orientalize refined olfactories.

We might spin out this article to twice its length, but the impatience of our readers, jealous of amusement when they look for instruction, admonishes us to end. Turkey is a bone of contention to the snarling powers of Europe. Its end is not far distant, and will be abrupt. Political physicians daintily feel the pulse of the "sick man;" hourly whisper his death; but, however agreed on the prognosis, are by no means as harmonious with regard to the treatment. Indeed, we hazard the uncharitable opinion that more than one incline to the heroic remedy adopted by Hazael toward his master. In view of this dark future, we shall never repent having jotted down the fireside prejudices and inner life of a nation doomed to lose its nationality and existence; just as the naturalist carefully labels here a rib, there a condyle of the now extinct Dodo.

